# Bibliography

**Published:** 1903-07-15

**Authors:** 


### BIBLIOGRAPHY.

"REGIONAL Anatomy of the Head and Neck," by Drs. William T. and Corinne B. Eckley, of the faculty of the medical and dental departments of the University of Illinois, is a new work published by Lea Bros. & Co. The text is very good and the illustrations, many of them being in colors, are very well selected. The text is largely made up of descriptive reference to possible surgical procedures, and, as a hand-book leading up to surgical operations, it should be very useful to students and practitioners as well. It however does not completely supply the requirements of the dental student for a course in descriptive anatomy. Many regions of the body are of as much importance to the dental student as bone and muscular tissue of the head and face. The brain and the related nervous organs and tissues should all be familiar to the dentist. The anatomy and physiology of the vital organs of the chest and abdomen should be as familiar to the dental practitioner as they are to the medical practitioner. The extensive use of general and local anesthetics, systemic anodynes and narcotics make it obligatory that the anatomy and physiology of the tissues should be well known to the dental practitioner.

As a guide to such surgical procedures as are liable to come within the sphere of the dental practitioner the book should be valued very highly. A considerable list of pointed quiz questions follows each chapter, and this ought to increase the value of the work as a teaching hand-book. We cheerfully commend the book to teachers and students, as we have no doubt but that it will meet many of the difficulties attending the study of this subject.

A NEW book called "Syphilis in Dentistry" is just out of the press of E. H. Colgrove, of Chicago. It comes from the pen of L. B. Baldwin, M. D., of Chicago, and Ezra R. Larned, M. D., of the same place. The book is dedicated "to our brothers in arms," the members of the dental profession, with the earnest hope that it may help in the recognition and alleviation of one of the greatest diseases of mankind. The title and the above dedicatory paragraph do not give a fair impression of the contents of the book. It is a carefully expressed resume of the important diagnostic manifestations of this dreadful disease, with a clear presentation of the possibilities of its being transferred from one patient to another by careless dental manipulations. The book is also well illustrated with colored plates of affected oral tissues which should be very helpful in enabling dentists to recognize its characteristic lesions. The book is small but sufficiently comprehensive for all practical purposes, and should be in the library of every dentist. Price $1.50.

